# Combining immune checkpoint inhibitors with thoracic radiotherapy enhances outcomes in advanced non-small-cell lung cancer: a real-world study

**DOI:** 10.3389/fonc.2025.1611528

**Published:** 2025-08-06

**Authors:** Yao Zou, Yichong Chen, Xiaojuan Zhou, Youling Gong, Yong Xu, Bingwen Zou, Feng Peng, Meijuan Huang, You Lu, Yongmei Liu

**Affiliations:** ^1^ Division of Thoracic Tumor Multimodality Treatment, Cancer Center, West China Hospital, Sichuan University, Chengdu, China; ^2^ West China Hospital Sichuan University, Meishan Hospital, Meishan, China; ^3^ Department of Oncology, Meishan People’s Hospital, Meishan, China

**Keywords:** immune checkpoint inhibitors, thoracic radiotherapy, non-small cell lung cancer, real-world study, survival outcomes

## Abstract

**Background:**

We aimed to evaluate the efficacy of thoracic radiotherapy (TRT) combined with immune checkpoint inhibitors (ICIs) in patients with advanced non-small-cell lung cancer (NSCLC) in real-world clinical settings and identify predictive subgroups that may benefit most from this approach.

**Methods:**

We retrospectively reviewed the medical records of patients with advanced NSCLC who were treated with ICIs at West China Hospital from January 2015 to May 2022.

**Results:**

A total of 302 patients with advanced NSCLC were included in this study. Among them, 54.3% (164/302) received ICIs in combination with TRT and were assigned to the TRT+ICIs group, while 45.7% (138/302) received ICIs alone and were assigned to the ICIs-only group. The median overall survival (OS) was significantly longer in the TRT+ICIs group (34.7 months) than in the ICIs-only group (27.1 months; P = 0.016). Additionally, the 24-month and 36-month OS rates were notably higher in the TRT+ICIs group (63.7% and 49.0%, respectively) than in the ICIs-only group (55.1% and 16.2%). Subgroup analysis of OS between the TRT+ICIs and ICIs-only groups identified factors associated with improved survival, including male sex, former smoking, Eastern Cooperative Oncology Group (ECOG) performance status 0–1, stage IIIb–c, high albumin level, and low neutrophil-to-lymphocyte (NLR) level. Multivariate analysis identified receipt of TRT, programmed death-ligand 1 (PD-L1) expression < 1%, PD-L1 ≥ 50%, and NLR as statistically significant independent prognostic factors for OS (P < 0.05). The combination treatment was well-tolerated, with an acceptable safety profile.

**Conclusion:**

Our findings suggest that adding TRT to immunotherapy improves survival outcomes in patients with advanced NSCLC.

## 1 Introduction

Immune checkpoint inhibitors (ICIs) have revolutionized the standard treatment strategies for patients with advanced and metastatic non-small cell lung cancer (NSCLC). ICIs, such as anti-programmed cell death protein 1 (PD-1) and programmed cell death ligand 1 (PD-L1), have been demonstrated to prolong overall survival (OS) in patients with metastatic NSCLC and have been approved as first-line systemic therapy for advanced NSCLC (1–3). However, the efficacy of ICIs is limited by both primary and acquired resistance (4). Preclinical studies have demonstrated that combining radiotherapy (RT) with ICIs offers synergistic benefits, including RT-induced tumor debulking, triggering immunogenic cell death, releasing tumor-associated antigens, activating tumor-associated dendritic cells, remodeling immunosuppressive tumor microenvironments, and modulating immune checkpoint molecule upregulation (5–8). Recently, an increasing number of clinical trials have explored the efficacy of combining ICIs with RT for NSCLC. Notably, the phase III PACIFIC (9)and GEMSTOM 301 (10) trials established the combination of RT and ICIs as a treatment modality for unresectable, stage III NSCLC. A phase I/II trial demonstrated that pembrolizumab, with or without concurrent RT, did not significantly affect the objective response rates (ORR) or progression-free survival (PFS) in patients with metastatic NSCLC (11). Numerous studies have suggested that combining RT and ICIs may benefit NSCLC treatment, though some have reported conflicting results (12, 13). These discrepancies are likely attributable to patient heterogeneity and variations in the modalities of combination therapy. Therefore, we aimed to evaluate the clinical outcomes of combining thoracic radiotherapy (TRT) and ICIs in patients with advanced NSCLC in real-world clinical practice and identify subgroups that may benefit most from this approach.

## 2 Materials and methods

### 2.1 Patients

**Figure 1:**
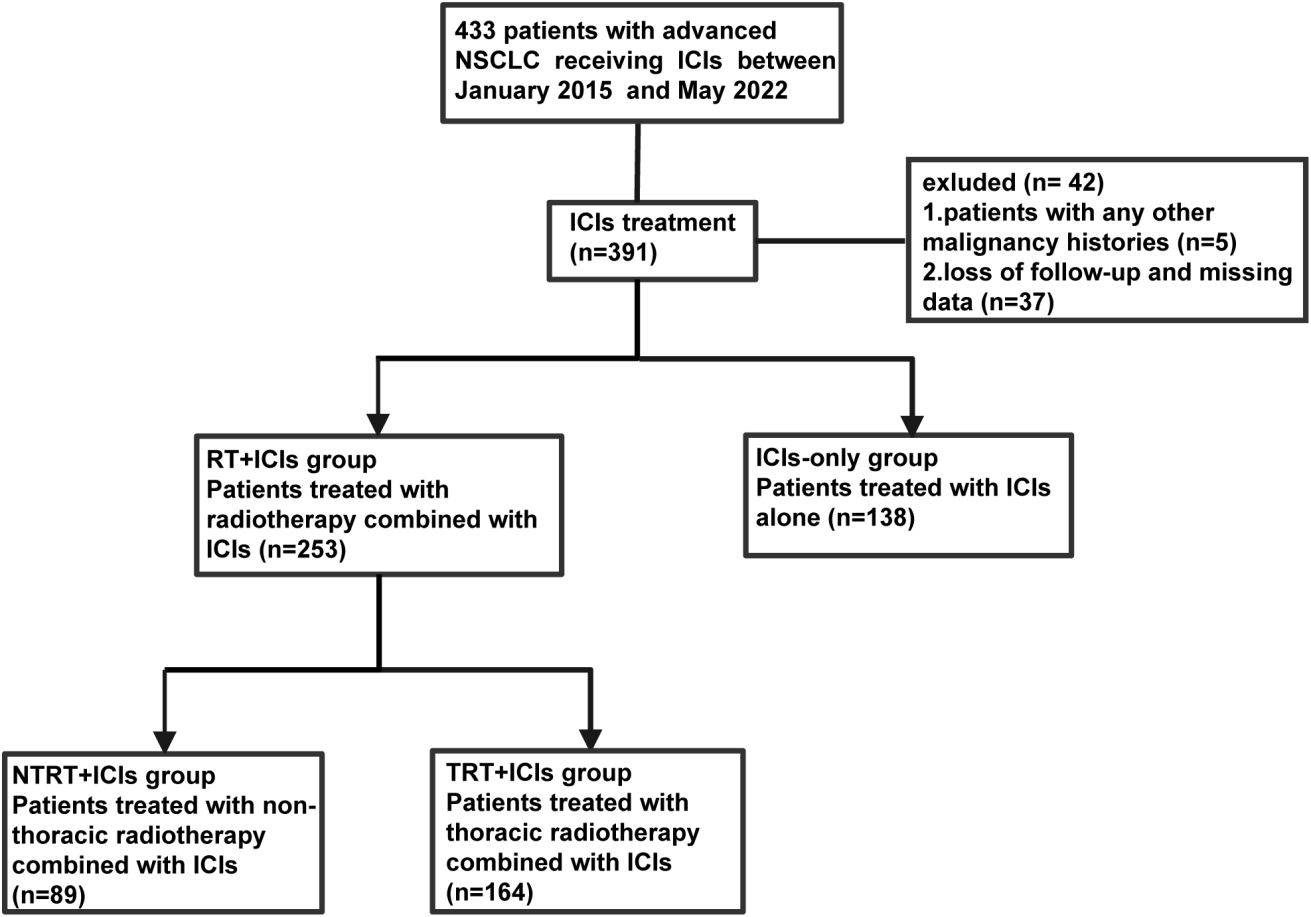
The flow chart of patient selection.

The medical records of patients diagnosed with advanced NSCLC and treated with ICIs, with or without TRT, at West China Hospital from January 2015 to May 2022 were included in this retrospective study. The inclusion criteria were as follows (1): patients were pathologically or cytologically confirmed to have stage IIIb–IV advanced NSCLC [according to the American Joint Committee on Cancer’s Cancer Staging Manual, 8th edition (14)] (2); patients received at least one dose of ICIs, with or without TRT, including definitive, palliative radiotherapy, and recurrence after definitive radiotherapy. The exclusion criteria were as follows (1): patients with a history of other malignancies (2); loss of follow-up or missing data; and (3) patients who received only RT from sites other than TRT (**Figure 1**).

The enrolled patients were categorized into two groups based on whether they received TRT. Patients who received ICIs combined with TRT were assigned to the TRT+ICIs group, while those who received only ICIs without RT were assigned to the ICIs-only group. The collected data included baseline patient demographics, pathology, stage at the initiation of ICI treatment, Eastern Cooperative Oncology Group (ECOG) performance status, PD-L1 expression, epidermal growth factor receptor (EGFR), anaplastic lymphoma kinase (ALK), proto-oncogene receptor tyrosine kinase (ROS1) aberration status, prior lines of systemic treatment, number of involved organs, albumin level at the initiation of ICIs, neutrophil-to-lymphocyte ratio (NLR) at initiation of ICIs, immunotherapy regimens, RT details, and follow-up data. This study was approved by the Ethics Committee of West China Hospital.

### 2.2 Treatments

The systemic immunotherapies used included pembrolizumab, nivolumab, durvalumab, atezolizumab, sintilimab, camrelizumab, tislelizumab, and penpulimab. The ICI regimens comprised monotherapy, combination with chemotherapy, or co-administration with a vascular endothelial growth factor receptor inhibitor. Patients in the TRT+ICIs group received a median of six cycles of immunotherapy (range: 1–39), whereas those in the ICIs-only group received a median of five cycles (range: 1–32).

Regarding the sequence of ICIs and RT, our study included both synchronous and sequential treatment approaches. For patients who received multiple courses of TRT, the course closest to the initiation of ICI therapy was selected for subsequent analysis. Patients received daily fractions ranging from 1.8 to 3 Gy, undergoing 10 to 30 fractions of conventional fractionated radiotherapy (CFRT) or 3 to 10 fractions of stereotactic body radiotherapy (SBRT), with a dose of 5.0 to 10.0 Gy per fraction for target organs. The biologically effective dose (BED) was calculated using the formula: BED = n * d * (1+ d ÷ [α/β]), where n = number of fractions, d = dose per fraction, and 10 is the assumed alpha/beta ratio for NSCLC tumors (15, 16). All patients received intensity-modulated RT or volumetric-modulated arc therapy, with a median BED dose of 60.0 Gy10 (range: 27.3 Gy10–180 Gy10). Adverse effects (AEs) were evaluated according to the National Cancer Institute Common Terminology Criteria for Adverse Events, version 4.03 (NCI CTCAE v4.03).

### 2.3 Statistical analysis

Ordinal variables were analyzed using the Mann–Whitney U test, while categorical variables were analyzed using the chi-square test, with a predetermined alpha level of 0.05 for statistical significance. All continuous parameters were examined for homogeneity of variance using Levene’s test, and comparisons were made using Student’s t-test. If the distribution significantly deviated from the normal, the Mann–Whitney U test was used. Albumin levels and NLR were categorized into two groups based on their median values. The primary endpoint of this study was the effect of TRT combined with ICI therapy on OS, which was measured from the start of ICI treatment to the time of death or censoring at the last follow-up. The Kaplan–Meier method was used to plot survival curves and estimate median OS. Differences in OS were compared using the log-rank test. In the subgroup analyses, the effect of adding TRT to ICIs on OS was assessed among the pre-set subgroups using Cox proportional hazards models, with results presented in a forest map. Bonferroni correction was adopted to enhance the credibility of the results. Univariate and multivariate analyses were performed using Cox proportional hazards regression models. Statistical significance was set at P < 0.05. All statistical analyses were performed using IBM SPSS Statistics (version 27.0; Armonk, NY, USA) and R (version 4.1.1; Vienna, Austria).

## 3 Results

### 3.1 Baseline characteristics

A total of 302 patients with advanced NSCLC who met the inclusion criteria were included in this study. As of the cut-off date, November 10, 2022, the median follow-up time was 26.6 months (95% confidence interval [CI]: 24.6–28.6). The patients were categorized into two groups based on whether they received TRT. Ultimately, 54.3% (164/302) of the patients were enrolled in the TRT+ICIs group, while 45.7% (138/302) were in the ICIs-only group. Baseline patient demographics and treatment characteristics of the two groups are summarized in **Table 1**. Although the baseline characteristics were generally well-balanced between the two groups, several notable differences were observed. Specifically, a greater proportion of patients in the TRT+ICIs group than in the ICIs-only group received ICIs as second-line or later-line therapy, were treated with ICIs monotherapy, and showed a higher NLR at the start of treatment. In contrast, the ICIs-only group had a higher proportion of never-smokers. Furthermore, more patients in the ICIs-only group received chemotherapy in combination with ICIs.

**Table 1:** Baseline demographics and treatment characteristics of TRT+ICIs group and ICIs-only group.

Parameters		ICIs-only (n=138)	TRT combined ICIs (n=164)	P-value
Age	≤61 (n, %)	63 (45.7)	83 (50.6)	0.391
	>61 (n, %)	75 (54.3)	81 (49.4)	
Sex	Male (n, %)	113 (81.9)	140 (85.4)	0.414
	Female (n, %)	25 (18.1)	24 (14.6)	
Treatment line of ICI	1 (n, %)	100 (72.5)	94 (57.3)	0.006
	≥2 (n, %)	38 (27.5)	70 (42.7)	
ECOG performance status	0-1 (n, %)	133 (96.4)	154 (93.9)	0.325
	≥2 (n, %)	5 (3.6)	10 (6.1)	
Smoking status	Never (n, %)	61 (44.2)	10 (6.1)	<0.001
	Current (n, %)	17 (12.3)	67 (40.9)	
	Former (n, %)	60 (43.5)	87 (53.0)	
Pathology	Adenocarcinoma (n, %)	76 (55.1)	72 (43.9)	0.151
	Squamous cell carcinoma (n, %)	52 (37.7)	76 (46.3)	
	Others/Mixed (n, %)	10 (7.2)	16 (9.8)	
Stage of disease	III (IIIb-c) (n, %)	31 (22.5)	52 (31.7)	0.074
	IV (n, %)	107 (77.5)	112 (68.3)	
EGFR/ALK/ROS1 aberration status	Positive (n, %)	9 (6.5)	8 (4.9)	0.338
	Negative (n, %)	92 (66.7)	122 (74.4)	
	Unknown (n, %)	37 (26.8)	34 (20.7)	
PD-L1%	<1% (n, %)	21 (15.2)	19 (11.6)	0.059
	1-49% (n, %)	28 (20.3)	49 (29.9)	
	≥50% (n, %)	24 (17.4)	47 (28.7)	
	Unknown (n, %)	65 (47.1)	49 (29.9)	
ICIs modalities	Monotherapy (n, %)	37 (26.8)	78 (47.6)	<0.001
	Chemotherapy combined (n, %)	91 (65.9)	71 (43.3)	
	VEGFR combined (n, %)	3 (2.2)	7 (4.3)	
	three modes combined (n, %)	7 (5.1)	8 (4.9)	
Albumin Mean (95%CI)		39.801 (39.137-40.466)	40.507 (39.929-41.085)	0.297
NLR Median (95%CI)		4.269 (3.681-4.858)	5.270 (4.556-5.984)	0.005
No. of involved organs	1-2 (n, %)	100 (72.5)	118 (72.0)	0.921
	≥3 (n, %)	38 (27.5)	46 (28.0)	

TRT, thoracic radiotherapy; ICIs, immune checkpoint inhibitors; ECOG, Eastern Cooperative Oncology Group; EGFR, epidermal growth factor receptor; ALK, anaplastic lymphoma kinase; ROS1, ROS1 proto-oncogene receptor tyrosine kinase; PD-L1, programmed cell death 1 ligand 1; VEGFR, vascular endothelial growth factor receptor; NLR, neutrophil-lymphocyte ratio.

### 3.2 Efficacy

**Figure 2:**
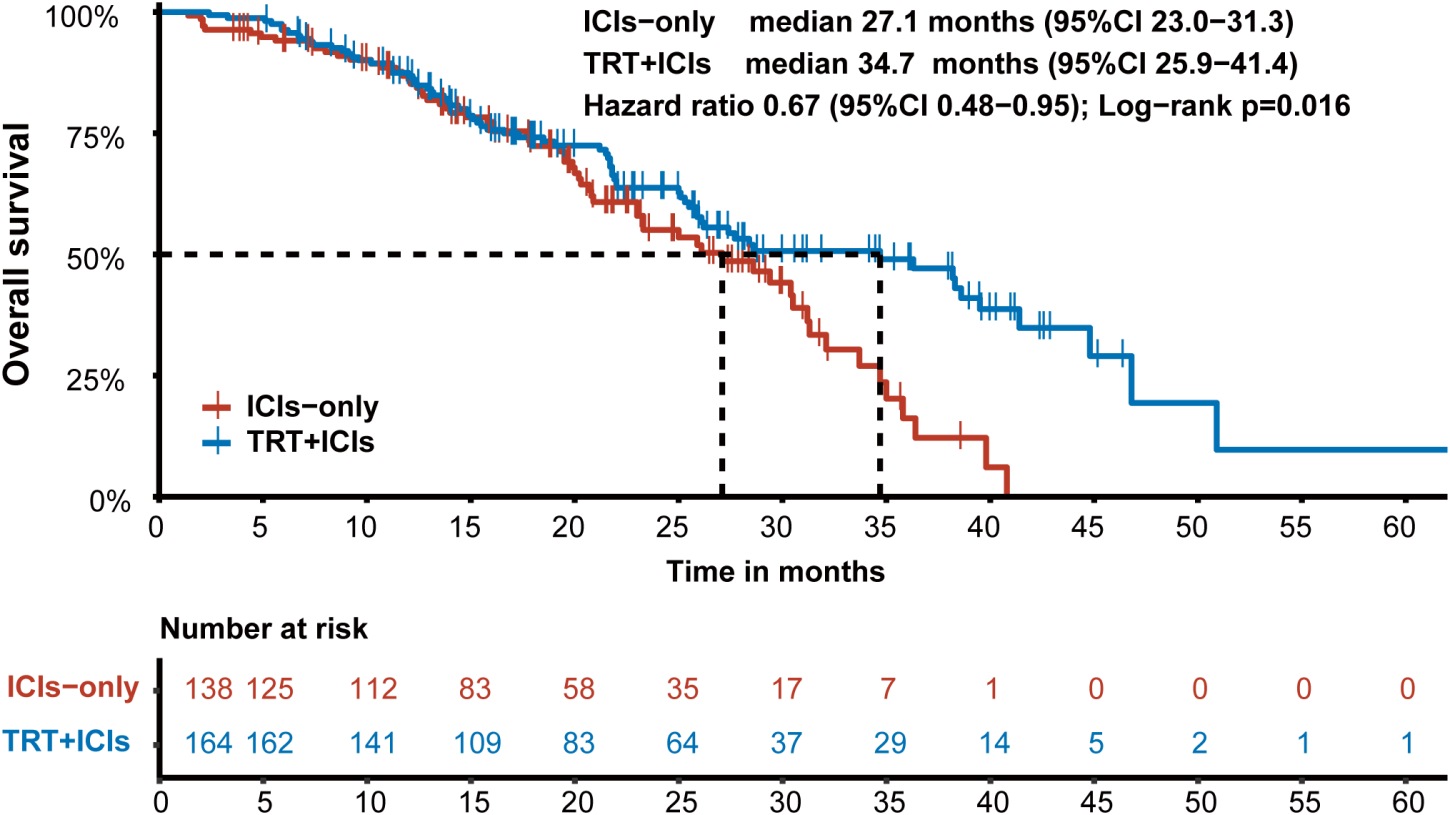
Kaplan-Meier survival curve of overall survival in the TRT+ICIs group and the ICIs-only group.

In the entire cohort, 45.7% (138/302) of patients died, with a median OS of 28.6 months (95% CI: 25.9–34.7). In the TRT+ICIs group, 44.5% (73/164) of patients reached the endpoint, while 47.1% (65/138) of patients in the ICIs-only group died. The median OS was significantly longer in the TRT+ICIs group (34.7 months) (95% CI: 25.9–41.4) than in the ICIs-only group (27.1 months) (P = 0.016) (95% CI: 23.0–31.3; **Figure 2**). Notably, the 24-month and 36-month OS rates were higher in the TRT+ICIs group at 63.7% (95% CI: 56.1%–72.4%) and 49.0% (95% CI: 40.3%–59.6%) than in the ICIs-only group, which had rates of 55.1% (95% CI: 45.8%–66.2%) and 16.2% (95% CI: 7.5%–34.8%), respectively.

**Figure 3:**
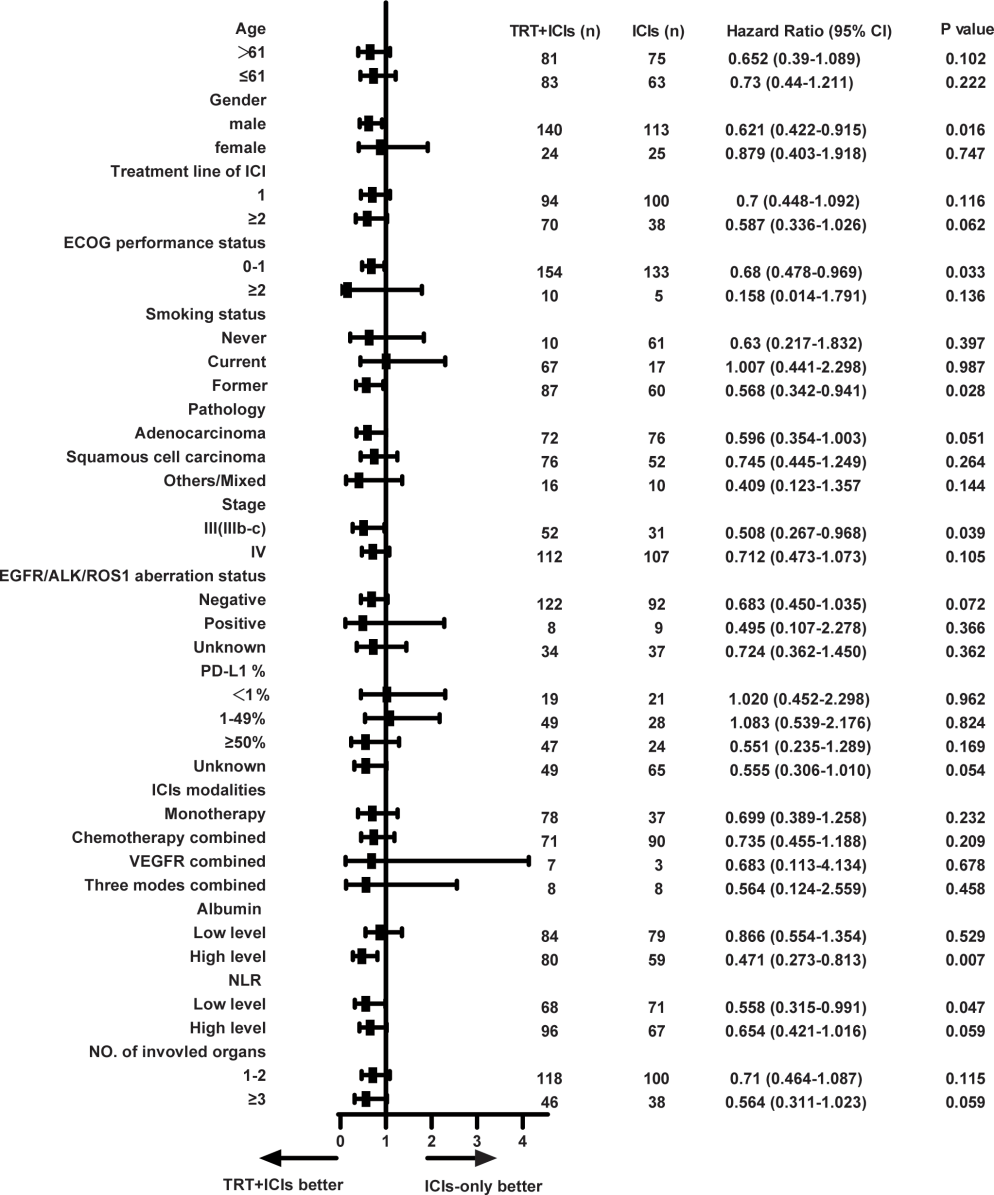
Forest plot of subgroup analysis on overall survival. TRT, thoracic radiotherapy; ICIs, immune checkpoint inhibitors; ECOG, Eastern Cooperative Oncology Group; EGFR, epidermal growth factor receptor; ALK, anaplastic lymphoma kinase; ROS1, ROS1 proto-oncogene receptor tyrosine kinase; PD-L1, programmed cell death 1 ligand 1; VEGFR, vascular endothelial growth factor receptor; NLR, neutrophil-lymphocyte ratio. * Exploratory subgroup analyses shown without multiplicity adjustment. All subgroup analyses became non-significant following Bonferroni correction (adjusted α = 0.0038).

We then conducted an exploratory subgroup analysis of OS between the two groups (**Figure 3**). The TRT+ICIs group demonstrated superior outcomes in men (hazard ratio [HR], 0.621; 95% CI, 0.422–0.915; P = 0.016), patients with an ECOG performance status of 0–1 (HR, 0.68; 95% CI, 0.478–0.969; P = 0.033), former smokers (HR, 0.568; 95% CI, 0.342–0.941; P = 0.028), patients with stage IIIb-c diseases (HR, 0.508; 95% CI, 0.267–0.968; P = 0.039), patients with high albumin levels (HR, 0.471; 95% CI, 0.273–0.813; P = 0.007), and patients with low NLR (HR, 0.558; 95% CI, 0.315–0.991; P = 0.047). Then, we applied Bonferroni correction (α = 0.05/13 subgroups = 0.0038) to all subgroup comparisons. As noted, this stringent adjustment rendered all comparisons non-significant.

Univariate survival analysis was performed to assess the association between OS and clinical characteristics, including age, sex, smoking status, prior lines of systemic treatment, ECOG performance status, pathology, stage, EGFR/ALK/ROS1 aberration status, PD-L1 expression, TRT, ICI modalities, number of involved organs, albumin level, and NLR. The results indicated that receiving TRT (HR, 0.655; 95% CI, 0.463–0.926; P = 0.017), PD-L1 < 1% (P = 0.003), PD-L1 ≥ 50% (HR, 0.348; 95% CI, 0.200–0.606; P < 0.001), albumin level (HR, 0.663; 95% CI, 0.471–0.934; P = 0.019), and NLR (HR, 1.600; 95% CI, 1.132–2.260; P = 0.008) were significant prognostic factors for OS (**Table 2**). Factors with P < 0.05 in the univariate analysis were included in the multivariate analysis. Multivariate analysis revealed that only receiving TRT (HR, 0.694; 95% CI, 0.485–0.994; P = 0.046), PD-L1 < 1% (P = 0.021), PD-L1 ≥ 50% (HR, 0.413; 95% CI, 0.235–0.727; P = 0.002), and NLR (HR, 1.528; 95% CI, 1.070–2.183; P = 0.020) remained statistically significant independent prognostic factors for OS (P < 0.05), as detailed in **Table 2**.

**Table 2:** Univariate and multivariable analysis of overall survival in the TRT+ICIs group and the ICIs-only group.

Parameters	Univariate OS		Multivariable OS	
	HR (95%CI)	P-value	HR (95%CI)	P-value
Age (years)				
≤61	–			
>61	1.130 (0.802-1.592)	0.485		
Sex				
Male	–			
Female	1.271 (0.843-1.917)	0.253		
Treatment line of ICI				
1	–			
≥2	1.036 (0.734-1.461)	0.842		
ECOG performance status				
0-1	–			
≥2	0.786 (0.321-1.921)	0.597		
Smoking status				
Never	–	0.576		
Current	0.794 (0.499-1.263)	0.329		
Former	0.774 (0.616-1.435)	0.940		
Pathology				
Adenocarcinoma	–	0.242		
Squamous cell carcinoma	1.335 (0.939-1.900)	0.108		
Others/Mixed	1.346 (0.723-2.503)	0.349		
Stage of disease				
III (IIIb-c)	–			
IV	0.877 (0.602-1.275)	0.491		
EGFR/ALK/ROS1 aberration status				
Negative	–	0.237		
Positive	1.311 (0.625-2.751)	0.474		
Unknown	1.385 (0.934-2.054)	0.105		
PD-L1%				
<1%		0.003		0.021
1-49%	0.657 (0.396-1.090)	0.104	0.725 (0.433-1.214)	0.222
≥50%	0.348 (0.200-.606)	<0.001	0.413 (0.235-0.727)	0.002
Unknown	0.635 (0.394-1.024)	0.062	0.667 (0.412-1.078)	0.098
Treatment group				
ICIs	–			
TRT + ICIs	0.655 (0.463-.926)	0.017	0.694 (0.485-0.994)	0.046
ICIs modalities				
Monotherapy	–	0.201		
Chemotherapy combined	1.387 (0.969-1.987)	0.074		
VEGFR combined	1.483 (0.589-3.732)	0.403		
three modes combined	1.905 (0.859-4.224)	0.113		
Albumin				
Low level	–			
High level	0.663 (0.471-.934)	0.019	0.773 (0.544-1.099)	0.152
NLR				
Low level	–			
High level	1.600 (1.132-2.260)	0.008	1.528 (1.070-2.183)	0.020
No. of involved organs				
1-2	–			
≥3	1.308 (0.917-1.866)	0.139		

TRT, thoracic radiotherapy; ICIs, immune checkpoint inhibitors; ECOG, Eastern Cooperative Oncology Group; EGFR, epidermal growth factor receptor; ALK, anaplastic lymphoma kinase; ROS1, ROS1 proto-oncogene receptor tyrosine kinase; PD-L1, programmed cell death 1 ligand 1; VEGFR, vascular endothelial growth factor receptor; NLR, neutrophil-lymphocyte ratio.

**Figure 4:**
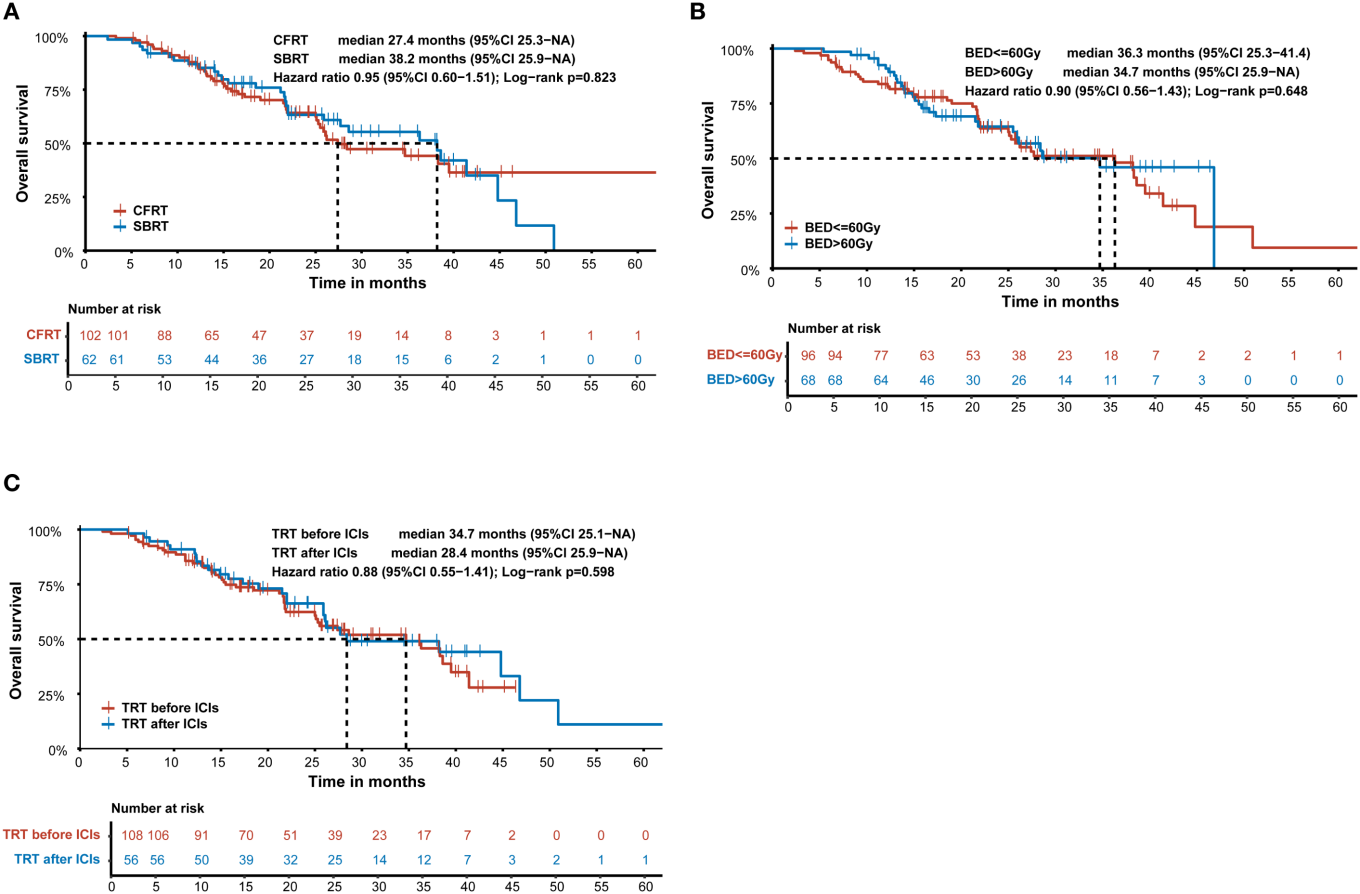
Kaplan-Meier survival curve of overall survival according to **(A)** SBRT and CFRT in the TRT+ICIs group. **(B)** the dose of TRT in the TRT+ICIs group. **(C)** the sequence between TRT and ICIs in the TRT+ICIs group. SBRT, stereotactic body radiotherapy; CFRT, conventional fractionated radiotherapy; BED, biologically effective dose; TRT, thoracic radiotherapy; ICIs, immune checkpoint inhibitors.

To investigate the optimal treatment combining TRT with ICIs, we conducted an exploratory subgroup analysis of OS in the TRT+ICIs group. Various TRT parameters were assessed. As illustrated in **Figure 4A**, patients who received SBRT had a higher median OS (38.2 months) (95% CI: 25.9–NA) than that of patients receiving CFRT (27.4 months) (95% CI: 25.3–NA). However, this difference was not statistically significant (P = 0.823). Additionally, we evaluated the impact of the TRT dose on OS by grouping patients based on BED equivalent doses of 60 Gy10. To examine the influence of the sequence between TRT and ICIs on prognosis, patients were stratified into two groups: those who received TRT before ICIs and those who received ICIs before TRT. Kaplan–Meier survival curves for the TRT dose and treatment sequence are presented in **Figures 4B, C**. However, no statistically significant differences were observed between these subgroups.

### 3.3 Treatment-related adverse events

The treatment-related AEs are summarized in **Table 3**. Among the 302 patients, 216 (71.5%) experienced at least one AE potentially related to therapy—121 (73.8%) in the TRT+ICIs group and 95 (68.8%) in the ICIs-only group (P = 0.343). The most common AEs were myelosuppression (41 [25.0%] in the TRT+ICIs group vs. 28 [20.3%] in the ICIs-only group; P = 0.331) and nausea (31 [18.9%] in the TRT+ICIs group vs. 30 [21.7%] in the ICIs-only group; P = 0.541). Overall, 24 (7.9%) patients experienced grade 3 or higher AEs (15 [9.1%] in the TRT+ICIs group and 9 [6.5%] in the ICIs-only group; P =0.401). The most frequent grade 3 or higher AEs included myelosuppression (5 [3%] in the TRT+ICIs group vs. 4 [2.9%] in the ICIs-only group; P > 0.999), pneumonitis (4 [2.4%] in the TRT+ICIs group vs. 2 [1.4%] in the ICIs-only group; P = 0.841), and transaminitis (3 [1.8%] in the TRT+ICIs group vs. 2 [1.4%] in the ICIs-only group; P > 0.999).

**Table 3:** Treatment-related adverse events.

AE	All Patients (n=302), n (%)		TRT+ICIs group (n=164), n (%)		ICIs-only group (n=138), n (%)		P-value	
	Any Grade	Grade ≥ 3	Any Grade	Grade ≥ 3	Any Grade	Grade ≥ 3	Any Grade	Grade ≥ 3
Any adverse event	216 (71.5)	24 (7.9)	121 (73.8)	15 (9.1)	95 (68.8)	9 (6.5)	0.343	0.401
Fatigue	16 (5.3)	0 (0.0)	8 (4.9)	0 (0.0)	8 (5.8)	0 (0.0)	0.722	–
Pain	1 (0.3)	0 (0.0)	0 (0.0)	0 (0.0)	1 (0.7)	0 (0.0)	0.457	–
Nausea	61 (20.2)	0 (0.0)	31 (18.9)	0 (0.0)	30 (21.7)	0 (0.0)	0.541	–
Myelosuppression	69 (22.8)	9 (3.0)	41 (25.0)	5 (3.0)	28 (20.3)	4 (2.9)	0.331	>0.999
Pneumonitis	24 (7.9)	6 (2.0)	20 (12.2)	4 (2.4)	4 (2.9)	2 (1.4)	0.003	0.841
Transaminitis	7 (2.3)	5 (1.7)	4 (2.4)	3 (1.8)	3 (2.2)	2 (1.4)	>0.999	>0.999
Dyspnea	2 (0.7)	1 (0.3)	1 (0.6)	1 (0.6)	1 (0.7)	0 (0.0)	>0.999	>0.999
Dermatitis	10 (3.3)	2 (0.7)	4 (2.4)	2 (1.2)	6 (4.3)	0 (0.0)	0.548	0.502
Thyroid dysfunction	22 (7.3)	0 (0.0)	12 (7.3)	0 (0.0)	10 (7.2)	0 (0.0)	0.981	–
Myocarditis	3 (1.0)	1 (0.3)	0 (0.0)	0 (0.0)	3 (2.2)	1 (0.7)	0.188	0.457
Renal dysfunction	1 (0.3)	0 (0.0)	0 (0.0)	0 (0.0)	1 (0.7)	0 (0.0)	0.457	–

The median onset time of pneumonitis in the entire cohort was 82 days (95% CI: 29.408–134.592) after the initiation of ICIs, with a median duration of 32 days (95% CI: 24.645–39.355). In the TRT+ICIs group, pneumonitis occurred at a median of 82 days (95% CI: 13.400–150.600) after ICIs initiation and 88 days (95% CI: 55.130–120.870) after TRT initiation, with a median duration of 36 days (95% CI: 29.070–42.930). Given the limited number of patients with pneumonitis in the ICIs-only group (n = 4), separate calculations were not performed.

## 4 Discussion

In our study, the combination of TRT and ICIs significantly prolonged OS and enhanced the 24-month and 36-month OS rates in patients with advanced NSCLC compared with ICIs alone. This highlights the efficacy of this combination strategy for treating advanced NSCLC. A secondary analysis of the KEYNOTE-001 trial showed that patients who had previously received radiotherapy exhibited longer PFS (4.4 months vs. 2.1 months, P = 0.019) and OS (10.7 months vs. 5.3 months, P = 0.026) than did those who did not, with an acceptable safety profile (17). Theelen et al. performed a secondary analysis of the MD Anderson Cancer Center (phase 1/2) and Pembrolizumab and Radiotherapy (phase 2) trials, where the thorax was the most common site targeted by radiotherapy (18). The combination of pembrolizumab and radiotherapy demonstrated a higher OS rate than that of pembrolizumab alone (19.2 months vs. 8.7 months, P = 0.0004), and PFS was significantly improved in the combination group (9.0 months vs. 4.4 months, P = 0.045). Additional radiotherapy significantly enhanced the out-of-field response rates (abscopal response rate: 41.7% vs. 19.7%, P = 0.0039; abscopal disease control rate: 65.3% vs. 43.4%, P = 0.0071). Moreover, neither of the two aforementioned trials achieved the predefined primary endpoints (11, 19). The phase III PACIFIC trial (9) demonstrated that the consolidation therapy with durvalumab following platinum-based concurrent chemoradiotherapy significantly improved PFS (P < 0.0001) and OS (P = 0.00251) in patients with unresectable stage III NSCLC. In conclusion, our findings are consistent with the current research, suggesting that the combination of RT with ICIs has considerable potential for the treatment of advanced NSCLC.

Although the clinical characteristics were not perfectly balanced between the two groups, a greater proportion of patients in the TRT+ICIs group received ICIs as second-line or later-line therapy, were treated with ICIs monotherapy, and showed a higher NLR at the start of treatment. According to the KEYNOTE-001 trial, enrolled patients with confirmed locally advanced or metastatic NSCLC received pembrolizumab (20). Treatment-naive patients demonstrated superior outcomes, with a median OS of 22.3 months (95% CI 17.1-32.3) compared to 10.5 months (95% CI 8.6-13.2) in previously treated patients. The 5-year OS rates were 23.2% and 15.5% for treatment-naive and previously treated cohorts, respectively. A retrospective cohort study by Heyward et al. analyzing SEER-Medicare data (2013–2019) from 17,681 individuals found that ICI plus chemotherapy showed significantly reduced mortality risk compared to ICI monotherapy in the first-line treatment setting (21). A Meta-analysis included data from 17 Phase III clinical trials involving 10,283 patients and demonstrated that immunotherapy significantly improves survival in lung cancer patients regardless of smoking status (never-smokers, former, or current smokers), with no significant interaction effects observed between treatment outcomes and smoking history (22). Ksienski et al. found that higher NLR significantly correlated with shorter OS in stage III NSCLC patients treated with durvalumab (23). Similarly, Lin et al. observed that increased NLR predicted worse OS in NSCLC patients receiving neoadjuvant therapy followed by surgical resection (24). All the aforementioned studies consistently demonstrated that the TRT+ICI group had poorer predicted survival outcomes. However, contrary to these expectations, the OS in the TRT+ICIs group was significantly longer than that in the ICIs-only group, providing further evidence for the effectiveness of this combined treatment strategy.

Preclinical evidence indicates that the combination of RT and ICIs produces synergistic effects through multiple mechanisms involving radiation-induced tumor cell killing that generates an *in situ* vaccine effect, enhances antigen presentation and dendritic cell activation, reprograms the immunosuppressive tumor microenvironment, and modulates immune checkpoint molecule expression (5, 6, 8, 25, 26). This study further confirms the therapeutic efficacy of the combined treatment.

The subgroup analysis of OS between the TRT+ICIs and ICIs-only groups identified factors associated with improved survival, including male sex (27), former smoking, ECOG performance status 0–1 (23, 28), stage IIIb-c (9, 10), high albumin level (29), and low NLR level (23, 28, 30). These findings almost align with those of previous studies, supporting the validity of our results. Only former smoking demonstrated a survival benefit inconsistent with the aforementioned meta-analysis results (22). This discrepancy may relate to the limited sample size in this study. In addition, the subgroup findings should be interpreted cautiously rather than definitive conclusions, particularly given non-significance after multiplicity correction. These results require prospective validation in adequately powered studies.

In the univariate survival analysis, TRT, PD-L1 expression, albumin level, and NLR were statistically significant. However, in the multivariate analysis, only TRT, PD-L1 expression, and NLR were independent predictors of prognosis. A significant association with OS was observed only in patients with PD-L1 < 1% and ≥ 50%. At the baseline level, the absolute proportion of PD-L1 ≥ 50% in the TRT + ICI group was indeed higher than that in the ICIs-only group (28.7% vs. 17.4%). However, there was no statistically significant difference between the two groups (P = 0.059). Moreover, in the multivariate analysis, the regression model was constructed to adjust for other confounding factors. This adjustment strategy ensures the reported protective effect of PD-L1 ≥50% is independent of baseline group differences. Patients in the PD-L1 ≥ 50% group were more likely than those in the PD-L1 < 1% group to benefit from treatment with ICIs, which is consistent with previous findings (3). No significant association was observed for the PD-L1 1-49% group, potentially due to the limited sample size. Ksienski et al. reported that elevated NLR was significantly associated with shorter OS in patients with stage III NSCLC treated with durvalumab (23).

Few studies have evaluated the optimal parameters for RT. In our study, we categorized RT techniques primarily into CFRT and SBRT. Our findings suggest that both CFRT and SBRT may enhance immunological efficacy. In the PACIFIC trial (9), patients received consolidation therapy with durvalumab following platinum-based concurrent chemoradiotherapy, with a typical CFRT dose of 60–66 Gy delivered in 30–33 fractions. A previous retrospective study concluded that SBRT (P = 0.013) and concurrent RT combined with ICIs (P = 0.002) were significantly associated with improved outcomes (31). Li et al. demonstrated that both CFRT (n = 75) and SBRT (n = 42), as well as intrathoracic RT (n = 76) and extrathoracic RT (n = 43), when combined with immunotherapy, could enhance survival (27). However, their study was limited by its relatively small sample size. Verma et al. reported that TRT in combination with ICIs is safe for patients, regardless of the different RT techniques and fractionation schedules (32).

In our study, no statistically significant difference was observed in the overall rate of treatment-related AEs between the TRT+ICIs and ICIs-only groups. Similarly, the incidences of grade 3 or higher AEs were comparable between the two groups. Although the overall incidence of pneumonitis was significantly higher in the TRT+ICIs group (12.2% vs. 2.9%, P = 0.003) than in the ICIs-only group, the majority of cases were mild to moderate (grade 1 or 2). Importantly, the incidence of clinically significant grade 3 or higher pneumonitis did not differ substantially between the two groups (2.4% vs. 1.4%; P = 0.841). Collectively, these findings suggest that the safety profile of thoracic radiotherapy combined with immunotherapy is well-tolerated and acceptable.

However, this study has some limitations. First, it was a retrospective, single-center study, which may have introduced some selection bias. Second, the PD-L1 expression status was not available for some patients. Third, in clinical practice, differentiating between radiation pneumonitis and immune-related pneumonitis remains a significant diagnostic challenge due to overlapping radiographic features, frequent concurrent administration of corticosteroids and limitations in retrospective study. Consequently, this study was virtually impossible to accurately distinguish between these two types of pneumonitis. Further well-designed randomized controlled trials are necessary to investigate the synergistic effects of RT and ICIs.

## 5 Conclusion

Our study supports the efficacy and safety of combining thoracic radiotherapy with immunotherapy in patients with advanced NSCLC. Additionally, this study delineates specific patient characteristics that may predict a favorable response to the proposed combination therapy, thereby providing a foundation for future research.

## Data Availability

The original contributions presented in the study are included in the article/supplementary material. Further inquiries can be directed to the corresponding author.
